# Complete Genome Sequence of Serotype III *Streptococcus agalactiae* Sequence Type 17 Strain 874391

**DOI:** 10.1128/genomeA.01107-17

**Published:** 2017-10-19

**Authors:** Matthew J. Sullivan, Brian M. Forde, Darren W. Prince, Deepak S. Ipe, Nouri L. Ben Zakour, Mark R. Davies, Gordon Dougan, Scott A. Beatson, Glen C. Ulett

**Affiliations:** a School of Medical Science, and Menzies Health Institute Queensland, Griffith University, Gold Coast, Australia; b Australian Infectious Diseases Research Centre and School of Chemistry and Molecular Biosciences, The University of Queensland, St. Lucia, Australia; c The Wellcome Trust Sanger Institute and The Department of Medicine, University of Cambridge, Cambridge, United Kingdom

## Abstract

Here we report the complete genome sequence of *Streptococcus agalactiae* strain 874391. This serotype III isolate is a member of the hypervirulent sequence type 17 (ST-17) lineage that causes a disproportionate number of cases of invasive disease in humans and mammals. A brief historical context of the strain is discussed.

## GENOME ANNOUNCEMENT

*Streptococcus agalactiae*, originally isolated as a causative agent of bovine mastitis (1), is a commensal of the human gastrointestinal and urogenital tracts of up to 30% of healthy adults (2). *S. agalactiae* is an opportunistic pathogen that causes sepsis, meningitis, pneumonia, and soft tissue infections, including urinary tract infection. The changing epidemiology of invasive disease due to *S. agalactiae* has highlighted an increasing incidence of infection in immunocompromised and elderly individuals (3). *S. agalactiae* strain 874391 (former strain number 24) (4, 5) is a human vaginal isolate previously studied in the context of pathogenomics (6–8); surface antigen structure (9); adhesion to, invasion, and killing of macrophages and epithelial cells (10–14); and urogenital tract colonization (15–17). *S. agalactiae* 874391 is of the hypervirulent sequence type 17 (ST-17) lineage that comprises homogenous serotype III clones that are associated with a disproportionately high number of cases of invasive neonatal disease, particularly meningitis (18–20). It is likely that the ST-17 *S. agalactiae* lineage originated from a bovine source (21).

DNA extraction and whole-genome sequencing were performed as follows. For Illumina sequencing, *S. agalactiae* 874391 genomic DNA was isolated using methods previously described (22). The DNA was used to generate 100-bp paired-end reads using the Illumina HiSeq 2000 platform at the Wellcome Trust Sanger Institute, United Kingdom. For Pacific Biosciences (PacBio) sequencing, DNA was isolated using the UltraClean microbial DNA isolation kit (Mo Bio Laboratories). Single-molecule real-time (SMRT) sequencing was performed on an RS-II machine (Pacific Biosciences, CA, USA) using P6-C4 chemistry at The University of Melbourne, Australia. The sequencing provided 477× coverage (1.17-Gb sequence, 68,825 reads, 17,033-bp mean read length).

For sequence analysis, PacBio sequence read data were assembled *de novo* using Canu version 1.3 (23). Following assembly, the genome was polished using Illumina sequencing data to resolve single nucleotide insertion and deletion errors associated with homopolymer tracts, generating a complete circular genome of 2,153,937 bp with a GC content of 35.5%. Detection of methylation signatures was carried out using the SMRT analysis package version 2.3.0. Annotation of the 2.15-Mb genome was performed using Prokka version 1.12 (24) and the NCBI Prokaryotic Genome Annotation Pipeline. Annotated features include 2,157 genes with 2,023 coding sequences (CDS), 21 ribosomal RNAs (rRNAs), 80 transfer RNAs (tRNAs), 3 noncoding RNAs (ncRNAs), 32 pseudogenes, and 1 clustered regularly interspaced short palindromic repeat (CRISPR) array. Annotation using the Rapid Annotation Subsystem Technology (RAST) server (25) showed that of the 2,023 CDS, 53% of the genes covered subsystem features. Of these, 68 were associated with virulence and 10 were associated with phages and prophages. Additionally, gene networks were linked to carbohydrate metabolism (*n* = 231), protein metabolism (*n* = 261), cell wall and capsule (*n* = 139), and resistance to antibiotics and toxic compounds (*n* = 34), including β-lactams (*n* = 1), fluoroquinolones (*n* = 4), tetracyclines (*n* = 2), vancomycin (*n* = 5), and multidrug efflux (*n* = 3).

### Accession number(s).

The genome has been deposited in GenBank under accession no. CP022537 (PacBio BioProject no. PRJNA395243, BioSample no. SAMN07374522), BioSample no. SAMEA1324071 (Illumina BioProject no. PRJEB2837), and the European Nucleotide Archive (accession no. ERS086616 and ERR126909).
